# Strategies to Reduce Mortality Among Children Living With HIV and Children Exposed to HIV but Are Uninfected, Admitted With Severe Acute Malnutrition at Mulago Hospital, Uganda (REDMOTHIV): A Mixed Methods Study

**DOI:** 10.3389/fped.2022.880355

**Published:** 2022-06-24

**Authors:** Victor Musiime, Andrew Kiggwe, Judith Beinomugisha, Lawrence Kakooza, Josam Thembo-Mwesige, Sharafat Nkinzi, Erusa Naguti, Loice Atuhaire, Ivan Segawa, Willy Ssengooba, Jackson K. Mukonzo, Esther Babirekere-Iriso, Philippa Musoke

**Affiliations:** ^1^Department of Paediatrics and Child Health, School of Medicine, College of Health Sciences, Makerere University, Kampala, Uganda; ^2^Department of Research, Joint Clinical Research Centre, Kampala, Uganda; ^3^Makerere University Lung Institute, College of Health Sciences, Makerere University, Kampala, Uganda; ^4^Mwanamugimu Nutrition Unit, Directorate of Paediatrics and Child Care, Mulago National Referral Hospital, Kampala, Uganda; ^5^BSL 3 Mycobacteriology Laboratory, Department of Medical Microbiology, School of Biomedical Sciences, College of Health Sciences, Makerere University, Kampala, Uganda; ^6^Department of Pharmacology and Therapeutics, School of Biomedical Sciences, College of Health Sciences, Makerere University, Kampala, Uganda

**Keywords:** HIV, HIV exposed children, severe acute malnutrition, mortality, antibiotics, Africa

## Abstract

**Background:**

Children living with HIV (CLHIV) and children who are exposed to HIV but uninfected (CHEU) are at increased risk of developing malnutrition. Severely malnourished children have high mortality rates, but mortality is higher in CLHIV/CHEU. This study aims to investigate whether empiric use of an antibiotic with greater antimicrobial sensitivity (ceftriaxone) than standard-of-care (ampicillin plus gentamicin) will reduce mortality among CLHIV/CHEU admitted with severe acute malnutrition.

**Methods:**

This is an open label randomized controlled trial involving 300 children; 76 CLHIV and 224 CHEU. The participants are being randomized to receive 1 week of ceftriaxone (*n* = 150) or standard-of-care (ampicillin/gentamicin) (*n* = 150), in addition to other routine care. The trial's primary outcome is in-hospital mortality. Secondary outcomes are: length of hospitalization; weight-for-height, weight-for-age and height-for-age z-scores; and pattern/antimicrobial sensitivity of pathogens. In addition, 280 severely malnourished children of unknown serostatus will be tested for HIV at admission to determine the prevalence and factors associated with HIV-infection. Furthermore, all the CLHIV on LPV/r will each provide sparse pharmacokinetic (PK) samples to evaluate the PK of LPV/r among malnourished children. In this PK sub-study, geometric means of steady-state LPV PK parameters [Area Under the Curve (AUC) _0−12*h*_, maximum concentration (C_max_) and concentration at 12 h after dose (C_12h_)] will be determined. They will then be put in pharmacokinetic-pharmacodynamic (PK-PD) models to determine optimal doses for the study population.

**Discussion:**

This study will ascertain whether antibiotics with higher sensitivity patterns to common organisms in Uganda and similar settings, will produce better treatment outcomes. The study will also provide insights into the current pattern of organisms isolated from blood cultures and their antimicrobial sensitivities, in this population. In addition, the study will ascertain whether there has been a significant change in the prevalence of HIV-infection among children presenting with severe malnutrition in the WHO recommended option B plus era, while determining the social/structural factors associated with HIV-infection. There will also be an opportunity to study PK parameters of antiretroviral drugs among severely malnourished children which is rarely done, and yet it is very important to understand the dosing requirements of this population.

**Trial Registration:**

ClinicalTrials.gov, identifier: NCT05051163.

## Introduction

Children living with HIV (CLHIV) are prone to developing malnutrition owing to medical and social factors including: higher daily calorie and micronutrient requirements than children who are unexposed to and uninfected with HIV (CHUU), a high frequency of infections including diarrhea which is often persistent/chronic, reduced intake because of a poor appetite and oral sores, late presentation to hospital, delayed initiation of antiretroviral therapy (ART) and food insecurity in households affected by HIV (1). CHEU have also been observed to be at risk of malnutrition; a prospective study among infants in Zimbabwe reported that they had lower mean weight-for length during follow up than CHUU (2). Because malnourished children are at risk of death, approaches that reduce mortality among CLHIV and CHEU who develop severe malnutrition, need to be evaluated.

Severely malnourished children have a high case fatality rate when admitted; studies in the East African Countries of Kenya, Tanzania and Uganda have reported mortality rates of 18–28% (3–5). HIV infection was associated with mortality in Uganda (4); similar to what had been observed in an earlier meta-analysis of data from Sub Saharan Africa (6). At the Mwanamugimu Nutrition Unit, Mulago Hospital, Uganda, studies conducted between 2001 and 2004 showed that ~40% of children admitted with severe acute malnutrition were HIV-infected (7–9). The overall mortality at the unit was 24% (4); but in a sub-analysis of those with bacteraemia, mortality was higher among the CLHIV than CHUU (43.5 vs. 20.5%) (8). CHEU were not identified in this study. More recent studies, in 2013 and 2017, at the unit have reported an HIV prevalence among the severely malnourished children of 24 and 12%, respectively. In these studies, a much higher mortality was observed among the CLHIV (33 and 47%, respectively) than in the CHUU (5 and 22%, respectively) (4, 10). The mortality rate among the CHEU in the 2017 study was 36% (4). Mortality therefore was more similar in CHEU and CLHIV than in CHUU. Preliminary audits at the unit indicate that 80–90% of the deaths are due to severe pneumonia and/or acute diarrhea. Late presentation and late initiation of ART play a role in increased mortality risks in CLHIV, (11, 12) but that cannot explain raised mortality risks in CHEU.

The WHO recommends that severely malnourished children receive empiric treatment with antibiotics for presumed bacteremia. This is because the typical signs of infection like fever may not be present in malnourished children given their impaired immunological response (13). The 2013 guidelines for management of severely malnourished children recommend amoxicillin for children admitted with severe acute malnutrition with no apparent signs of infection and no complications. Those with complications should receive ampicillin for 2 days followed by amoxicillin for 5 days, plus gentamicin for the 7 days of ampicillin/amoxicillin (i.e., combination treatment); if there is no improvement in 48 h, chloramphenicol is added for 7 days (13). This is the current practice in Uganda (14). However, these recommended drugs may no longer be effective in Uganda and elsewhere due to high rates of observed antimicrobial resistance (7, 8, 15, 16). On the other hand, studies have reported favorable susceptibility patterns to ceftriaxone (7, 8, 16, 17). It is therefore important to investigate, in these settings, whether antibiotics with higher sensitivity scores to common organisms, such as ceftriaxone, will produce better treatment outcomes. It is also necessary to ascertain whether there has been a change in the pattern of organisms isolated from blood cultures and their antimicrobial sensitivities.

Studies in Tororo, Uganda and South Africa have suggested that malnutrition may have an impact on the bioavailability of antiretroviral drugs including lopinavir/ritonavir, which is currently an alternative drug in first and second-line ART regimens in Uganda (18). Sub-optimally dosed antiretroviral drugs in HIV-infected severely malnourished children may therefore contribute to the poor outcomes we observe (19, 20).

This study aims to investigate whether empirical use of an antibiotic with greater antimicrobial sensitivity (ceftriaxone) than standard of care (ampicillin plus gentamicin) will reduce mortality among CLHIV and CHEU admitted with severe acute malnutrition at Mwanamugimu Nutrition Unit, Mulago Hospital. Secondary objectives include: to compare the length of hospitalization, weight-for-height, weight-for-age and height-for-age z-scores between ceftriaxone vs. standard of care (ampicillin and gentamicin) treatment arms. These anthropometric indices and length of hospitalization are hypothesized to be affected by infection related morbidity which may be influenced by the antibiotics received. Other secondary objectives include: to ascertain the frequency of different bloodstream bacterial pathogens and their antimicrobial sensitivities; to ascertain the prevalence of, and factors associated with, HIV-infection among children admitted with severe acute malnutrition at Mwanamugimu Nutrition Unit; and to evaluate the pharmacokinetic (PK) parameters of LPV/r among severely malnourished CLHIV.

## Materials and Methods

### Study Design

The main study will be an open label randomized controlled trial among HIV-infected/HEU severely malnourished children admitted at Mwanamugimu Nutrition Unit. The children will be randomized to receive 1 week of ceftriaxone vs. standard-of-care (ampicillin plus gentamicin), both in addition to other routine care. The trial will be open-label because, it is difficult and potentially unethical to blind intravenous drug administration. Secondly, a cross-sectional evaluation will be done among severely malnourished children at admission; testing them for HIV to determine the prevalence of HIV-infection among severely malnourished children in the option-B-plus era. In addition, qualitative data will be collected from mothers of CLHIV to understand the sociocultural/system facilitators of HIV transmission. Thirdly, a PK sub study will be conducted within the clinical trial to evaluate the PK parameters of LPV/r among these severely malnourished children using sparse PK samples.

### Study Setting

Mwanamugimu Nutrition Unit at Mulago Hospital is a specialized center for management of malnutrition. It is the largest such center in Uganda and acts as a practicum site for universities in Uganda, including Makerere University. It consists of 3 in-patient (stabilization, transition and rehabilitation) wards, an outpatient therapeutic feeding center, a laboratory, a pharmacy and a kitchen. It is run by a team of 3 pediatricians, 2–6 postgraduate doctors at a time, 2–5 intern doctors, 1–2 intern nurses, 1 intern pharmacist, 25 nurses, 3 nursing assistants, 2 nutritionists, 4 nutrition assistants, and a laboratory technician. In 2021, 737 severely malnourished children were admitted to the unit^1^. It is a government owned and government run institution, so the services are designed to be provided at no cost to the patient/caregiver.

### Study Population

Three hundred children aged 1 month to 5 years (76 CLHIV and 224 CHEU) whose caretakers provide informed consent will be randomized to receive ceftriaxone (*n* = 150) or ampicillin plus gentamicin (*n* = 150). The 1 month to 5-year age group has been selected because it is the most affected by severe acute malnutrition and constitutes over 95% of admissions at Mwanamugimu Nutrition Unit. Also, the 1:3 ratio of CLHIV to CHEU in this sample is reflective of the current proportions of CLHIV and CHEU admitted at Mwanamugimu Nutrition Unit.

Three hundred randomized children provide >80% power to detect reductions in mortality from 28% (average across CLHIV and CHEU in preliminary audits at the unit) to 14% (50% relative reduction; absolute 14% improvement), allowing for 10% non-compliance/loss-to-follow-up (leaving hospital without official discharge) in each group. When half the participants have been enrolled (*n* = 150), the data safety monitoring board (DSMB) will review the preliminary data to determine if these estimates are appropriate. In addition, at least 280 severely malnourished children and their mothers will be tested for HIV at admission; any positive children will also be eligible to participate in the clinical trial. Two hundred eighty children provide 80% power to determine the prevalence of HIV-infection, using the prevalence of HIV-infection of 24% obtained in the probisam study in Mwanamugimu in 2013 (10), as a reference. The CLHIV in the clinical trial on LPV/r will take part in the pharmacokinetic sub-study providing 3 sparse PK samples each.

### Selection Criteria for Quantitative Data

#### Inclusion Criteria

i. CLHIV and CHEU aged 1 month to 5 years admitted at Mwanamugimu Nutrition Unit with severe acute malnutrition.
ii. For prevalence of HIV-infection sub-study, children presenting with severe acute malnutrition on admission at Mwanamugimu Nutrition Unit.
iii. For PK sub-study, the child should have been on lopinavir containing antiretroviral therapy for at least 2 weeks and should have been in hospital for at least 1 week; and on ready to use therapeutic feeds (RUTF).
iv. Parent or guardian provides informed consent, including consent for HIV testing.

#### Exclusion Criteria

a. Readmission for management of severe acute malnutrition.

For PK sub-study;

b. A child with documented poor adherence to antiretroviral therapy.
c. A child on anti – TB medication.
d. A child known to have vomited the drug on the sampling day.

### Selection Criteria for Qualitative Data

#### Inclusion Criteria

i. Mothers of CLHIV with severe acute malnutrition.
ii. Provision of informed consent.

#### Exclusion Criteria

a. Inability to undergo in-depth interviews, for such reasons as illness and unavailability.

### Study Procedures

All severely malnourished children and their mothers will have rapid HIV antibody tests on admission as per routine practice; at least 280 children will participate in the cross-sectional study. Children under 18 months of age with positive HIV results will have blood taken for DNA PCR. The children who test positive or whose mother's test positive (i.e., are CHEU) will be eligible for enrolment into the trial. Participant enrolment will be done between 9.00 am and 4.00 pm from Monday to Friday to allow for blood samples to be taken to the laboratories on the day of enrolment. HIV rapid testing will be done at the unit (Mwanamugimu); HIV DNA PCR at the Baylor Uganda center of excellence (COE) laboratory at the Mulago Hospital Complex. At enrolment, anthropometry and a physical examination will be done; resuscitation will be provided as needed and a blood sample will be taken off for blood culture. The blood culture and sensitivity will be done in the Microbiology laboratory at the Makerere University College of health Sciences (MakCHS). After all enrolment procedures have been done, children will be randomized to ceftriaxone vs. ampicillin/gentamicin. The children will then receive antibiotics as randomized for 1 week together with other routine care. At day 8 (on completion of study medication), the children will be evaluated to ascertain any events that may have occurred and document administration of the study medication. They will then be followed up until discharge/death whichever occurs first. ART naïve CLHIV will initiate ART during admission while ART-experienced children will continue their regimens; both groups will receive daily cotrimoxazole throughout follow-up. The eligible CLHIV on LPV/r (target number – 76) will have 3 sparse PK samples taken at least 2 weeks after ART initiation and at least 1 week after admission to hospital (after they have completed the stabilization phase of treatment); the samples will be taken over 2–3 days depending on what is physiologically possible for the children. The samples will be collected from the study population to cover three time points. The child will take an observed morning dose and then sampling will be done at 3 h, at 6 h and at 12 h (just before the evening dose). LPV concentrations in the plasma samples will be determined using ultra performance liquid chromatography with ultraviolet detection in the Department of Pharmacology and Therapeutics, MakCHS. All laboratories are on the same location on Mulago hill, within a 1 km radius. During admission, the mothers will be interviewed to ascertain sociocultural/ system factors related to antenatal care attendance, prior HIV testing, knowledge and access to PMTCT services, infant feeding practice and ART adherence (theirs and the children's as applicable). Some of the mothers (~20) will be asked to participate in in-depth interviews. These will be consecutively enrolled until data saturation. These interviews will be tape recorded, transcribed verbatim and then translated into English where applicable. Any adverse events that develop will be managed according to the treatment protocols of Mwanamugimu Nutrition Unit/ Mulago Hospital. The principal investigator will be notified of these events within 24 h, who will in turn inform the trial sponsor within 24 h; and the Research Ethical Committee (School of Medicine Research Ethical Committee at Makerere University) within 7 days; and Uganda National Drug Authority within 7 days, as per the local research guidelines.

The procedures, which will be performed by a study doctor, a study nurse and a study counselor trained on the study protocol, are summarized in the trial schema (Table 1).

**Table 1:** Redmothiv trial schema.

**Assessments/ procedures**	**Days in trial**									
	**Screening**	**Randomization (day1)**	**Daily until day 7**	**Day 8**	**At discharge or death**	**PK day 1**	**PK day 2**	**PK day 3**	**Qualitative study day**	**Acute event**
**Trial participation/ clinical care**										
Parent/guardian information	X									
Informed consent		X								
Cross-sectional study/ HIV testing	X									
Clinical assessment	X	X		X	X	X	X	X		
Enrolment form		X								
Mother's form		X								
Follow up form				X						
Routine care	X	X	X	X	X	X	X	X	X	X
Drug administration		X	X							
Blood culture		X								
PK sampling						X	X	X		
In depth interviews									X	
Death					X					
Discharge					X					
Serious adverse event										X

### Participant Randomization

Randomization lists will be generated by the Trial statistician, using a computer, so that the study clinical team remain blinded about the allocation of the next child to be enrolled. The study coordinator will arrange for randomization codes to be printed onto cards placed in opaque sealed envelopes, a system that has worked well in other trials in acutely sick children. The envelopes will be stored on the ward and will be numbered consecutively and opened in numerical order by the study doctor.

### Data Management and Statistical Analysis

A database will be designed to capture the patient data from the study tools. Data will be checked for completeness before entry into the database. The database will be backed up on a daily basis. The study tools for each patient will be put in separate folders and kept in lockable cabinets. The primary outcome will be in hospital mortality. Secondary outcomes will be: length of hospitalization (for those that are followed up till discharge); as well as weight-for-height, weight-for-age and height-for-age z-scores during follow up. The change in these anthropometric indices (z-scores) from admission to discharge will be determined, while controlling for the number of days of hospitalization. Another secondary outcome will be the distribution/antimicrobial sensitivity of pathogens.

Mortality (and other time-to-event data) will be compared between randomized groups using Kaplan Meier curves and Cox proportional hazards regression. WHO reference ranges will be employed to determine the anthropometric z-scores which will be analyzed using linear regression. Other categorical factors will be compared between randomized groups using chi-squared and exact tests, and continuous data using rank-sum tests. The statistical analyses will be performed in STATA version 15 (Stata Corporation, Houston, Texas).

Qualitative data from the in-depth interviews of the mothers will be analyzed using a content thematic approach, using *NVivo software*. We will enroll mothers and perform ongoing analysis until there is saturation of data.

For primary analysis in the PK sub study, sparse PK samples will be obtained from approximately 76 CLHIV. Using NONMEM version 7.3, PK-PD modeling and simulations will be performed to determine primary PK parameters, measures of plasma drug exposure and the dosing requirements.

### Ethical Considerations

The research protocol has been approved by the Makerere University School of Medicine Research Ethical Committee (REC. REF. 2020 - 165) and the Uganda National Council of Science and Technology (REF. HS1277ES). Regulatory approval was obtained from the Uganda National Drug Authority (NDA) (REF. CTC. 0168/2021). Administrative clearance was obtained from Mulago Hospital (REF. MHREC. 1942). All mothers of the participating children will provide written informed consent, using the consent documents in English and local 5 local languages (Luganda, Runyoro-Rutooro, Luo, Ateso and Swahili), as appropriate. The data collected will be kept under lock and key and saved in a secure database. Published patient data will not have names and confidentiality will be ensured throughout the study. All blood sampling done (including PK sampling) will be within recommended volumes taken during the specified period among malnourished children (21). Individual patient results will be provided to the Mwanamugimu Nutrition Unit staff for care as needed.

### COVID-19 Risk Management Plan

The trial is being implemented during the SARS-CoV-2 (COVID−19) pandemic. It is therefore imperative that efforts are made to protect the participants and the study staff from contracting the COVID-19 infection. Also, in case any of the participants or the study staff develops COVID-19 infection, they will be referred to the COVID-19 treatment unit of Mulago hospital for management. Personal protective equipment such as face masks, hand sanitizers and access to COVID-19 vaccination will be provided to study staff and participants, building on what is provided by Mulago hospital and the Uganda ministry of health. These measures are captured in an approved study risk management plan and standard operating procedures (SOPs). The plan will also include how the potential effects from the pandemic, such as slow recruitment due to reduced patient numbers in hospital, reduced workforce, lockdowns affecting movement, will be handled to ensure successful implementation of the study.

## Discussion

There is no published comparison of ceftriaxone vs. ampicillin/gentamicin (standard of care) in rates of mortality among CLHIV and CHEU admitted with severely acute malnutrition. This study provides the opportunity to evaluate it. This is important because infection-related mortality in this population is very high. It is also important to ascertain whether there has been a change in the pattern of organisms isolated from blood cultures and their antimicrobial sensitivities. In this study, ceftriaxone will be used empirically. Whilst it is possible that this could lead to adverse events such as diarrhea, skin rash and hypersensitivities, it is unlikely that these will occur more frequently than in CHUU or well-nourished children (13). There are also concerns regarding development of resistance, but benefits from reducing mortality may outweigh the risk of development of resistance. These risks and benefits however need to be formally evaluated.

In 2013, WHO recommended the “option B plus” strategy for prevention of mother to child transmission of HIV (PMTCT). Universal ART is recommended for pregnant women and nursing mothers living with HIV and the infants receive antiretroviral drug prophylaxis as part of comprehensive care (22). This not only has the potential to reduce HIV transmission to infants to <5% at 1 year, but could also improve the survival of CHEU (23, 24). Despite Uganda adopting the strategy in 2013, HIV-infected young infants continue to present to health units, and increasing numbers of CHEU are also presenting with poor outcomes when hospitalized (4). Therefore, ascertaining whether there has been a significant change in the prevalence of HIV-infection among children presenting with severe malnutrition in the option B plus era, while determining the social/structural factors associated with acquisition of HIV despite availability of PMTCT, will be useful as we aim to eliminate mother to child transmission of HIV.

Malnourished CLHIV have been reported to have poor outcomes on ART (11, 12); part of the explanation could be that the doses of antiretroviral drugs determined with well-nourished children may not be appropriate for the severely malnourished children who are known to have an altered physiology. For example, the drug levels in individuals are affected by the protein binding capacity of the drugs and it is known that malnourished children have low levels of protein (albumin) (25), implying that for the drugs that are highly bound to protein there would be a lot of free drug in malnourished children with a potential to cause an excess of adverse events. Examples of such drugs are lopinavir and ritonavir which are 98–99% bound to the blood plasma proteins albumin and alpha-1-acid glycoprotein (26). The Ugandan recommended alternative first line ART regimen for HIV infected children, is abacavir+lamivudine+lopinavir/ritonavir. Lopinavir/ritonavir (LPV/r) is also a key alternative drug in second-line regimens (18). In this project, we will investigate the pharmacokinetics of LPV/r among the severely malnourished children on ART, thereby providing an opportunity to test the hypothesis that severely malnourished children are ineffectively dosed according to the Ugandan guidelines, which does not differentiate well-nourished from malnourished children. It is possible that the severely malnourished children are exposed to toxic levels of free drug given the low level of albumin and drugs with high protein binding capacity. We will obtain sparse PK samples from the children while stable, likely in the second week of hospitalization, with the volumes of blood collected being those that are physiologically safe, such as those provided in a review of safe limits to volume by Howie SR (21). The study will also build on the work on PK and acceptability of LPV/r pellets in the CHAPAS 2 trial where bioequivalence between the pellets and syrup was demonstrated and the pellets were highly acceptable (27) and the follow on and ongoing implementation study of the LPV/r pellets (LIVING study) (28); as well as the LOLIPOP study evaluating the PK and acceptability of the 4 in 1 (abacavir+lamivudine+lopinavir/ritonavir) sprinkle formulation (29). Dolutegravir based regimens are becoming increasingly available and it is possible during the study period there are children that will be on dolutegravir. Dolutegravir PK will be considered among these children.

While the study will compare length of hospitalization and anthropometric z-scores between the two study arms as secondary outcomes, it will be difficult to control for social factors, which is a limitation of the study. Another limitation is that there may be children over the age of 18 months whose mothers are dead or unavailable. If these children test HIV negative, when their mothers were HIV infected, they will be taken as CHUU instead of CHEU, and will therefore be wrongly excluded from the study. On the other hand, a child over the age of 18 months may test HIV negative, while the mother tests HIV positive. Such a child will be taken as CHEU. However, the mother may have had a recent acquisition of HIV, and the child may not have necessarily been exposed to HIV. This child would wrongly be included in the study. We expect that children in these two scenarios will be very few. Furthermore, to obtain the sample size of 300, we made the assumption that the intervention would make a 50% relative reduction in mortality. While this is unusual, it is possible; on the other hand, a significantly smaller difference would result in a sample size that would make it logistically untenable to conduct the study.

The findings of this study in general will provide insights into designing protocols aimed at improving treatment outcomes, particularly reduced mortality, not just in CLHIV/CHEU but also overall mortality among severely malnourished children. Secondly, given that among CLHIV, the malnourished are at an increased risk of mortality (12), strategies that reduce mortality among severely malnourished CLHIV would result in an overall reduction in mortality among CLHIV.

## Ethics Statement

The studies involving human participants were reviewed and approved by School of Medicine Research Ethics Committee, College of Health Sciences, Makerere University. Written informed consent to participate in this study was provided by the participants' legal guardian/next of kin.

## Author Contributions

VM, WS, JM, and PM designed the study. JB, AK, LK, SN, IS, JT-M, EN, and LA are participating in the management of the study participants and data collection. VM, WS, JM, EB-I, and PM are supervising the management of the participants and data collection. All authors reviewed all versions and approved the final version of the manuscript.

## Funding

This study was funded by Makerere University Research and Innovations Fund (MakRIF) with a grant (Award No. MAK/DVCFA/113/20) from the Government of Uganda.

## Conflict of Interest

The authors declare that the research was conducted in the absence of any commercial or financial relationships that could be construed as a potential conflict of interest.

## Publisher's Note

All claims expressed in this article are solely those of the authors and do not necessarily represent those of their affiliated organizations, or those of the publisher, the editors and the reviewers. Any product that may be evaluated in this article, or claim that may be made by its manufacturer, is not guaranteed or endorsed by the publisher.

## Footnotes

^1^Mulago-National-Referral-Hospital. Hospital Records (2022). [Unpublished].
